# Ranibizumab for choroidal neovascularization secondary to pseudoxanthoma elasticum: 4-year results from the PIXEL study in France

**DOI:** 10.1007/s00417-017-3685-y

**Published:** 2017-05-10

**Authors:** Gérard Mimoun, Jean-Marc Ebran, Typhaine Grenet, Alain Donati, Salomon-Yves Cohen, Anne Ponthieux

**Affiliations:** 1 Centre ophtalmologique de l’école militaire, Paris, France; 2 University hospital of Angers, Angers, France; 3 University hôpital of Avicenne, Bobigny, France; 4 Ophthalmic Center of Laser and Medical Imaging, Paris, France; 5 Ophthalmic center, Melun, France; 6 University Hospital of Creteil, Creteil, France; 7 Novartis Pharma S.A.S, Rueil-Malmaison, France

**Keywords:** Angioid streaks, Choroidal neovascularization, Observational, Pseudoxanthoma elasticum, Ranibizumab

## Abstract

**Purpose:**

To evaluate the long-term effectiveness and safety of ranibizumab 0.5 mg in patients with choroidal neovascularization (CNV) secondary to pseudoxanthoma elasticum (PXE) in a real-world setting.

**Methods:**

A descriptive, observational, multicenter study in a retrospective and prospective cohort was conducted in France that included patients who had received at least one injection of ranibizumab 0.5 mg during the period October 2011 to October 2014, for CNV secondary to PXE. Eligible patients were identified by review of medical records or during routine consultations. The main objectives were to describe patient characteristics, assess changes in best-corrected visual acuity [VA, Early Treatment Diabetic Retinopathy Study (ETDRS) letters] over time, the number and reasons for ranibizumab treatment and overall safety.

**Results:**

Of the 72 enrolled patients (98 eyes) from 23 centers, 39 (54.2%) were male and mean [±standard deviation (SD)] age was 59.6 (±8.3) years. The mean VA was 64.6 letters at the first ranibizumab injection, which was maintained at the 1-year follow-up (64.7 letters). Thereafter, the mean VA was stable until the 4-year follow-up. At 4 years, the proportion of eyes with VA gain of ≥15 letters was 3/19 (15.8%) and stable VA (change between −15 and +15 letters) was 10/19 (52.6%). Mean (±SD) annual number of ranibizumab injections was 4.1 (±4.0), lower in the second versus first year. The most common reason for ranibizumab treatment was progression of neovascular activity (42.9%). No deaths or new safety findings were reported.

**Conclusions:**

In patients with CNV secondary to PXE, ranibizumab 0.5 mg resulted in stable VA over 4 years with a limited number of injections. Safety findings were consistent with the established safety profile of ranibizumab.

## Introduction

Pseudoxanthoma elasticum (PXE) is a rare, predominantly autosomal recessive, systemic disorder caused by mutations in the adenosine triphosphate-binding cassette subtype C number 6 (ABCC6) gene [1, 2]. It primarily affects the skin, eyes, and cardiovascular system [2]. The underlying pathology of PXE involves progressive calcification and fragmentation of elastic fibers in the affected tissues [2]. The global prevalence of PXE is estimated to be approximately 1 in 25,000 to 150,000 [1–3]. PXE has a higher female preponderance and affects approximately twice as many females than males [4].

PXE manifests mainly in the posterior segment of the eye as reticular macular dystrophy, angioid streaks (AS), drusen of the optic nerve, peau d’orange, and comet-like peripheral lesions [5, 6]. It is one of the most common causes of AS, accounting for 59%–87% of cases [7]. There is degeneration of the elastic fibers of the Bruch’s membrane in PXE, which calcify and fragment to form breaks in the Bruch’s membrane that are clinically evident as AS [8]. AS are asymptomatic in the earlier stages; however, in the later stages, they become symptomatic due to the development of choroidal neovascularization (CNV) in the subretinal space [1, 2]. CNV is the most severe complication of AS and develops in 72–86% of the eyes, more often bilaterally [7, 9–11]. If left untreated, it leads to central vision loss and legal blindness in over 50% of cases [1]. AS-associated CNV affects a relatively young and often active population, thus substantially affecting their quality of life [12].

Laser photocoagulation [13–15], verteporfin photodynamic therapy (vPDT) [16], and surgery [17, 18] have been explored in the past for treatment of CNV secondary to AS, albeit with little success due to the high rates of recurrence and progressive visual loss. Anti-vascular endothelial growth factor (VEGF) therapy has been used successfully for the treatment of CNV associated with age-related macular degeneration (AMD) [19, 20]. Likewise, treatment with anti-VEGF agents such as bevacizumab (Avastin®; Genentech/Roche) [21], and ranibizumab (Lucentis®; Genentech, South San Francisco, CA, USA; and Novartis Pharma AG, Basel, Switzerland) [22] has shown promise in CNV secondary to AS in several small studies with a short follow-up duration [23–28]; a recently published long-term retrospective study reinforces these short-term outcomes [29]. However, all these studies are in patients with AS-associated CNV in causes other than PXE. In AS-associated CNV secondary to PXE particularly, favorable visual outcomes over a long term with anti-VEGF therapy has been reported in few case reports and smaller studies [12, 23, 24, 30–34].

To date, no medical treatment is approved for the treatment of CNV secondary to PXE. Results from short case series of one to nine patients with CNV secondary to PXE treated with ranibizumab have been encouraging [24, 30, 32–34]. However, data from large sample size over a long term are unavailable. In France, ranibizumab was granted temporary reimbursement from October 2011 to October 2014, for the treatment of patients with CNV secondary to PXE. In this context, the PIXEL study was conducted to evaluate the long-term effectiveness and safety of ranibizumab 0.5 mg in patients with CNV secondary to PXE in a real-world setting.

## Methods

### Study design

PIXEL was a multicenter, descriptive, and observational study of a retrospective and prospective cohort conducted in real-world settings in France. Eligible patients were identified retrospectively by review of medical records or prospectively during routine consultations; the two methods were not mutually exclusive. A total of 5509 ophthalmologists across France were invited to participate to make the study as comprehensive as possible. The study protocol was designed to ensure inclusiveness by informing all French ophthalmologists regarding the study occurrence and requesting their participation through emails, letters, and phone calls. Patient participation was voluntary, and the ophthalmologists could prescribe treatment and follow-up with patients at their own discretion. The study was conducted in compliance with the Best Practices for Epidemiological Studies of April 2007. The study was approved by the Data Protection Advisory Committee and the National Commission on Informatics and Freedom. Informed consent was obtained from all individual participants included in the study.

### Patients

Patients who had received at least one injection of ranibizumab 0.5 mg in one eye since October 7, 2011 for CNV secondary to PXE and had provided signed informed consent (from legal guardians for minors) for participation were included in the study. Both eyes of a patient were included for analysis, if eligible. Administration of ranibizumab 0.5 mg treatment to patients with CNV secondary to PXE was authorized by the French Health Authority between October 2011 and October 2014.

### Data collection

Data were collected at different time points during routine treatment of the patients: at the time of diagnosis of CNV secondary to PXE, between diagnosis of CNV and the first ranibizumab injection, at the time of first ranibizumab injection, and during each routine visit after the first ranibizumab injection. A prospective follow-up visit was performed at 3, 6, 12, and 18 months from the time of obtaining the informed consent. Patient enrollment, follow-up, and data collection were terminated in October 2014.

### Study objectives

No primary objective was defined for the study. The objectives were as follows: (a) to describe demographic and clinical characteristics of the patients and eye(s), (b) to describe the functional and anatomical changes in the patients and eye(s) following ranibizumab treatment, (c) to assess the average annual number of ranibizumab injections, (d) to assess the reasons for re-administration, treatments other than ranibizumab, and reasons for their administration, (e) to assess the annual average number of patient follow-up visits, and (f) to assess safety and tolerability of ranibizumab.

### Endpoints

Functional and anatomical changes: The endpoints for assessing the functional changes included mean best-corrected visual acuity [VA, Early Treatment Diabetic Retinopathy Study (ETDRS) letters] at each visit from the initiation of ranibizumab treatment and proportion of eyes with change in VA. The proportion of visually impaired patients was also evaluated according to the visual impairment definition of the World Health Organization (WHO): corrected VA between ≥20 and ≤60 ETDRS letters (1/20 and 3/10) in the better eye. Visual loss was defined as the VA corrected to a range of ≥20 and ≤65 ETDRS letters in the better eye (i.e. 1/20 and 4/10). Stable VA was defined as a change in VA between −15 and +15 ETDRS letters. The time to bilateral impairment was the duration between the first symptom in the eye/VA decline/diagnosis of new vessels/ date of first ranibizumab injection (in the order of preference as indicated) in the first and the second eye impaired. The anatomical outcomes included proportion of eyes with change in CNV, CNV leakage on fluorescein angiography, and retinal hemorrhages and change in central retinal thickness (CRT) over 4 years from the time of ranibizumab initiation. The stabilization or regression of CNV lesion was considered as disease remission.

Treatment exposure*:* The mean annual number of ranibizumab injections was calculated from the first injection as 365.25 × number of ranibizumab injections/ (date of the last visit - date of the first injection). Similarly, the mean annual number of visits was calculated from the first ranibizumab injection as 365.25 × number of consultations/(date of the last visit - date of the first ranibizumab injection). The follow-up duration was calculated from the first ranibizumab injection in the first ranibizumab-treated eye.

Safety and tolerability*:* The safety and tolerability were assessed by recording adverse events (AEs), non-serious and serious AEs occurring from the first injection of ranibizumab 0.5 mg throughout the study period. AEs were coded as per the Medical Dictionary for Regulatory Activities (MedDRA) and were summarized as total number of AEs, proportion of patients with at least 1 AE including at least 1 AE by system organ class and preferred term as well.

### Statistical analyses

All analyses were conducted on “patients” and “eyes” analyses populations. The patient analysis population comprised patients with ocular complications secondary to PXE who had received at least one injection of ranibizumab 0.5 mg between October 7, 2011 and October 6, 2014 and had provided a signed informed consent form. For each patient, the first “affected” and the first “ranibizumab-treated” eyes were identified. The investigator reported the data from the first affected eye for the patient analysis population. A patient was considered to have a “bilateral” impairment if the pathology affected both the eyes (two affected eyes). The eyes analysis population comprised the ranibizumab-treated eyes from the patient analysis population. An eye was considered as treated if it had received at least one injection of ranibizumab between October 7, 2011 and October 6, 2014. The impaired eyes that were not treated with ranibizumab during the study inclusion period were excluded from the study. Therefore, in case of bilateral impairment, only the eye treated with ranibizumab was included in the study. The safety analysis population included the same number of patients as that in the patient analysis population.

The study objectives were purely descriptive. Descriptive analyses of the characteristics of all included patients were presented. The missing data were excluded while calculating the percentage for VA outcomes. Quantitative variables were described as mean, standard deviation (SD), median, and range, and qualitative variables in terms of absolute frequency and percentage by method.

The statistical analyses were performed using SAS software® version 9.2.

## Results

### Doctor participation, patient disposition, baseline demographics, and disease characteristics

A total of 211 responses were received from the invited 5509 ophthalmologists, of which 70 (33.2%) agreed to participate in the study. Of these, 23 (32.9%) ophthalmologists from 23 centers enrolled at least one patient. The primary reason given for non-participation by 80.9% (114/141) of the ophthalmologists was lack of PXE patients, which was expected given the rarity of the disorder. Other reasons for non-participation by ophthalmologists included lack of time (7, 5.0%), general non-participation in studies (5, 3.5%), lack of interest in the study (3, 2.1%), or “other” (7, 5.0%).

A total of 75 patients were enrolled in the study. Of the enrolled patients, three were excluded from the study. The reasons for exclusion of the patients were treatment outside of the study reference period in two patients and documented wrong injection during the evaluation period for one patient. Thus, a total of 72 patients were included in the patient analysis population. The mean follow-up duration was 39.2 (±27.3) months and the mean number of visits was 25.9 (±21.9).

A total of 150 eyes corresponding to the 75 patients were enrolled. Of these, 98 (65.3%) eyes were treated with ranibizumab within the study reference period (October 2011–October 2014) and included in the eyes analysis population. The mean follow-up duration was 36.5 (±26.0) months. Thirteen eyes had retrospective follow-up data for 6 years, but due to their small numbers, the data were analyzed only for the first 4 years following the first injection of ranibizumab.

A majority of the patient analysis population was male (39; 54.2%), and the mean age was 59.6 (±8.3) years at the time of consent for participation in the study. The mean age of patients at the onset of first symptoms in the first impaired eye was 55.5 (±10.6) years. At the time of initiation of ranibizumab treatment, 47 (65.3%) and 25 (34.7%) patients had one and both eyes impaired, respectively. The median duration of the onset of symptoms between the two eyes was 2 years, and mean time to diagnosis of CNV between the two eyes was 2.4 (±3.0) years. The mean VA at the time of ranibizumab initiation in the first impaired eye and contralateral eye was 65.4 (±19.4) and 57.5 (±27.3), respectively.

All eyes for which study data were available (*N* = 69) underwent eye examinations. The most commonly performed examinations were fundus ophthalmoscopy (91.3%), optical coherence tomography or fluorescein angiography (79.7% for each of the tests), and color or monochromatic fundus photography (73.9%). Autofluorescence and indocyanine green angiography were performed less frequently (49.3% and 34.8%, respectively). Approximately half of the patients (32, 44.4%) had non-ocular complications secondary to PXE of which a majority (27, 84.4%) had skin complications. Data for skin biopsy were available for only 24 (33.3%) patients at the time of diagnosis, of which two had a doubtful result and further information was missing for one. The remaining 21 (87.5%) patients had positive results. The reasons for low numbers of biopsies could include the presence of a characteristic PXE lesion, easily identifiable by experienced dermatologists, not requiring confirmation by skin biopsy or avoidance of the procedure due to its invasive nature or complexities involved in interpreting results. A high proportion of eyes (77.6%) had CNV, more frequently located juxtafoveally and subfoveally, at the initiation of ranibizumab treatment. At the time of diagnosis of ocular complications secondary to PXE, a high proportion of patients had ocular symptoms (90.3%, 65) and AS (91.7%, 66) in the first ranibizumab-treated eye. Other ocular characteristics of the patients and eyes analyses populations are shown in Table 1.

**Table 1:** Ocular characteristics (patients and eyes analyses populations) at the initiation of ranibizumab 0.5 mg treatment

Characteristic^a^	Patient analysis population *N* = 72		Eyes analysis population *N* = 98
	First treated eye *N* = 72	Contralateral eye *N* = 72	
Angioid streaks, n (%)	66 (91.7)	56 (77.8)	73 (74.5)
CNV leakage, n (%)	51 (70.8)	21 (29.2)	50 (51.0)
Retinal hemorrhages, n (%)	34 (47.2)	10 (13.9)	33 (33.7)
CNV, n%	64 (88.9)	30 (41.7)	76 (77.6)
Location	64	30	76
Subfoveal	24 (37.5)	12 (40.0)	25 (32.9)
Juxtafoveal	23 (35.9)	8 (26.7)	28 (36.8)
Extrafoveal	19 (29.7)	9 (30.0)	19 (25.0)
Unspecified	3 (4.7)	5 (16.7)	5 (6.6)
VA (ETDRS letters), mean (±SD)	65.4 (±19.4) (*n* = 54)	57.5 (±27.3) (*n* = 45)	–
CRT (μm), mean (±SD)	298.7 (±134.7)	256.7 (±79.8)	291.1 (±133.2)

^a^Patients with absent ocular characteristic and those with missing data are excluded

*CNV*, choroidal neovascularization; *CRT*, central retinal thickness; *ETDRS*, Early Treatment Diabetic Retinopathy Study; *SD*, standard deviation; *VA*, visual acuity

### Efficacy

#### Bilateral impairment after initiation of ranibizumab 0.5 mg (patient analysis population)

Bilateral impairment of eyes was observed in 29 (40.3%) patients at the time of the first ranibizumab injection and 38 (52.8%) at the 1-year follow-up, 48 (66.7%) each at the 2-year and the 4-year follow-up. The mean time between the first and the second eye to visual impairment and the onset of symptoms was 46.19 (±79.87) and 51.37 (±102.48) months, respectively.

#### Functional changes after initiation of ranibizumab 0.5 mg (eyes analysis population)

**Fig. 1:**
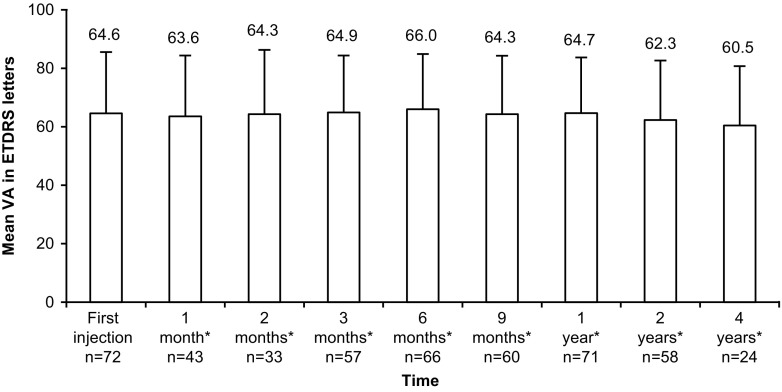
Mean VA at each visit from the time of first ranibizumab 0.5 mg injection until 4 years (eyes analysis population) *After the first injection of ranibizumab (± 1 week for 1 and 2 months, ± 2 weeks for 3 and 6 months, ± 1 month for 9 months, and ±2 months for all other time points). ETDRS, early treatment diabetic retinopathy study; VA, visual acuity

**Fig. 2:**
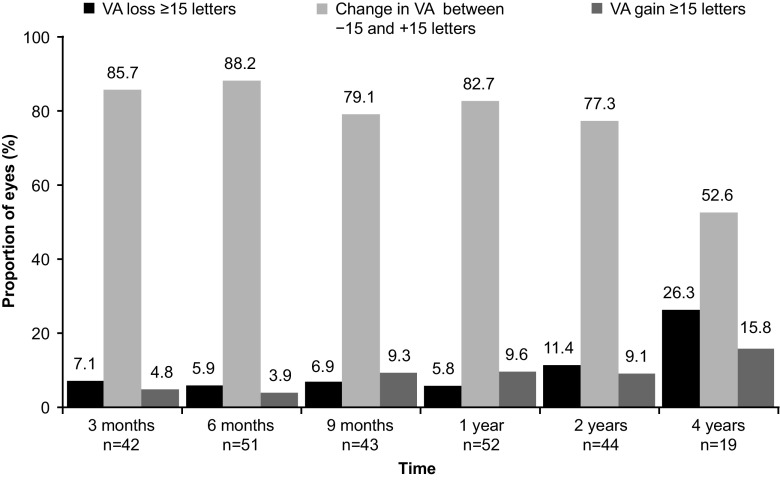
Proportion of eyes with change in VA after initiation of ranibizumab 0.5 mg treatment until 4 years (eyes analysis population) Stable VA, Change in VA between −15 and +15 ETDRS letters VA, visual acuity

The mean VA was 64.6 letters at the first ranibizumab injection, which was maintained at the 1-year follow-up (64.7 letters). Thereafter, VA remained stable until the 4-year follow-up (Fig. 1). The proportion of patients with a VA loss of ≥15 ETDRS letters, VA gain of ≥15 ETDRS letters or stable VA over 4 years is shown in Fig. 2. At the 4-year follow-up, 3/19 (15.8%) patients showed a VA gain of ≥15 letters and 10/19 (52.6%) had a stable VA.

A total of 29 patients had VA impairment after the first ranibizumab injection over the entire follow-up duration. However, VA improved in five patients at the end of the follow-up. The proportion of visually impaired patients (per the WHO definition) increased over time from 4.8% (*n* = 2) at 1 month to 21.7% (*n* = 5) by the 4-year follow up after the first ranibizumab injection. One patient was considered blind (VA <1/20 in both eyes) after 6 years of the first ranibizumab injection with a VA of 5 ETDRS letters in the left eye and 15 ETDRS letters in the right eye. Another patient with visual impairment did not remain blind over the entire study duration as the VA improved to 20 ETDRS letters at the last evaluation.

#### Anatomical changes after initiation of ranibizumab 0.5 mg (eyes analysis population)

**Fig. 3:**
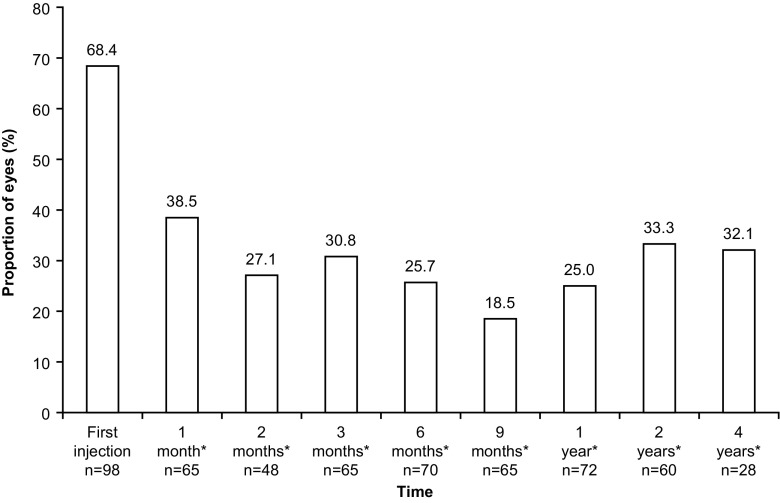
Proportion of eyes with choroidal neovascularization at each visit from the time of first ranibizumab 0.5 mg injection until 4 years (eyes analysis population) *After the first injection of ranibizumab (± 1 week for 1 and 2 months, ± 2 weeks for 3 and 6 months, ± 1 month for 9 months, and ±2 months for all other time points)

The proportion of eyes with CNV decreased from 68.4% (67/98) at the time of the first ranibizumab injection to 32.1% (9/28) at the 4-year follow-up (Fig. 3). CNV disappeared in 22.9% (11/48) of the eyes, 2 months following the first ranibizumab injection, and this proportion increased to one third at the 1-year (33.3%, 24/72), 2-year (30.0%, 18/60), and 4-year (32.1%, 9/28) follow-up. The proportion of eyes with presence of CNV leakage was 39.8% at the time of first injection and 14.3% at the 4-year follow-up. The proportion of eyes with retinal hemorrhages decreased from 26.5% at the first injection to 5.6% over 1 year, remained stable thereafter, and was 7.1% at the 4-year follow-up. Regardless of the time of evaluation, 95.9%, 74.5% and 60.2% of the eyes had CNV, CNV leakage and retinal hemorrhages, respectively.

The mean CRT in the eyes analysis population initially decreased from 293.4 (±126.1) μm at the time of the first ranibizumab injection to 247.1 (±49.7) μm at the 6-month follow-up and was subsequently stable with a mean CRT of 247.4 (±59.0) μm at the end of the 4-year follow-up.

#### Treatment exposure (eyes analysis population)

Treatment with ranibizumab 0.5 mg: The mean duration of ranibizumab treatment was 2.4 (±2.2) years. The mean duration between the first and the second ranibizumab injection was 114.8 (±204.4) days. The mean annual number of ranibizumab injections was 4.1 (±4.0); 4.1 (±2.3) in the first year and 2.7 (±2.3) in the second year. More than half of the eyes (56.5% [52/92]) received the first three ranibizumab injections in 4 months, 47.9% (45/94) in 3 months, and 12.6% (12/95) in 2 months.

**Fig. 4:**
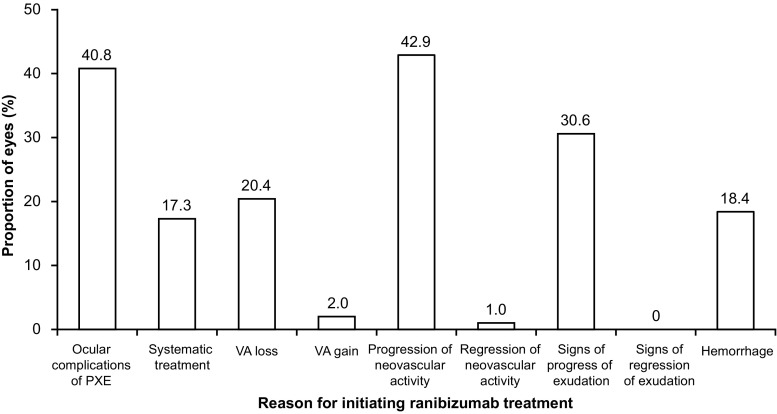
Reasons for initiating treatment with ranibizumab 0.5 mg (eyes analysis population) PXE, pseudoxanthoma elasticum; VA, visual acuity

The most common reasons for initiating ranibizumab treatment (not exclusive) were progression of neovascular activity in 42.9% eyes and ocular complications of PXE in 40.8% eyes (Fig. 4). The most frequent reasons for re-treatment with a second (58.2%) and a third (51.2%) injection were systematic treatment (25.5%) as per the dosage recommendation of ranibizumab label, and CNV progression and signs of exudation (38.4%) for the injections corresponding to the maintenance period.

##### Previous treatment

Thirty-five (35.7%) eyes had a history of previous treatment before ranibizumab initiation. Of these, 22 (22.4%) had received treatment with anti-VEGF other than ranibizumab, 17 (17.3%) had received vPDT, nine (9.2%) had received thermal laser photocoagulation, and five (5.1%) had received corticosteroids. The mean number of previous treatments received was 8.4 (±9.2) with anti-VEGF other than ranibizumab, 2.7 (±3.0) with vPDT, 1.7 (±0.8) with thermal laser photocoagulation and 1.0 (±0.0) with corticosteroids.

##### Concomitant treatment

Thirteen eyes received concomitant treatment, with initial monthly injections of ranibizumab, to arrest CNV progression. Of the total 75 concomitant treatments administered, 68 were with anti-VEGF other than ranibizumab, three each were vPDT and corticosteroids, and one was thermal laser photocoagulation.

### Safety (safety analysis population)

Overall, 24 AEs were reported in 14 (19.4%) patients, of which 14 were ocular AEs reported in 10 (13.9%) patients. The most frequent ocular AEs were ocular pain in three (4.2%) patients and decrease in VA in two (2.8%) patients. A total of 10 SAEs were reported in six (8.3%) patients. A 50-year old female patient, with a history of dyslipidemia, hypertension since 2011 and bilateral carpal tunnel syndrome and a family history of dyslipidemia, hypertension, type 2 diabetes and arterial hypertension, was hospitalized for cerebral ischemic infarction, suspected to be related to ranibizumab treatment. Magnetic resonance imaging (MRI) of the brain confirmed the presence of an ischemic infarct in the left pontine region. Before the event, she had received 17 injections of anti-VEGF agents (including 15 ranibizumab injections) and 11 injections of anti-VEGF agents (including nine ranibizumab injections) in the left and right eye, respectively. She was receiving concomitant treatment with rosuvastatin, telmisartan, acetylsalicylate lysine, candesartan, hydrochlorothiazide, and cholecalciferol. Following her recovery 1 week later, the patient restarted ranibizumab treatment and received nine injections of ranibizumab. Another patient, a 53-year old male, was hospitalized following a transient ischemic attack, which was considered not related to ranibizumab by the investigator. An ultrasound of the supra-aortic trunks and transcranial Doppler showed stage III stenosis of the end of the left internal carotid artery and occlusion of the right vertebral artery. A transesophageal and transthoracic echocardiogram showed a bicuspid aortic valve and dilatation of the thoracic aorta (52 mm). MRI of the brain revealed ischemia of the left lenticular nucleus. Arterial thromboembolic events pose a theoretical risk to patients receiving anti-VEGF therapy and were monitored as part of the ranibizumab risk management program. One SAE of endophthalmitis was suspected to be related to intravitreal injection technology. No death was reported in the study. The overall safety summary is presented in Table 2.

**Table 2:** Overall safety summary (safety analysis population)

Safety parameter	Patients, *N* = 72 n (%)	AEs^a^
Total AEs	14 (19.4)	24
Ocular AEs Eye pain Visual acuity decrease Amaurosis Cataract Conjunctival hemorrhage Eye irritation Eyelid oedema Macular oedema Retinal vascular disorder Blurred vision Vitreous detachment	10 (13.9) 3 (4.2) 2 (2.8) 1 (1.4) 1 (1.4) 1 (1.4) 1 (1.4) 1 (1.4) 1 (1.4) 1 (1.4) 1 (1.4) 1 (1.4)	14 3 2 1 1 1 1 1 1 1 1 1
Nervous system disorders Carotid stenosis Ischemic cerebral infarction Sciatica Transient ischemic attack Occlusion of the vertebral artery	3 (4.2) 1 (1.4) 1 (1.4) 1 (1.4) 1 (1.4) 1 (1.4)	5 1 1 1 1 1
Infections and infestations	2 (2.8)	2
Congenital, familial and genetic disorders	1 (1.4)	1
Injury, poisoning, and complications linked to the procedures	1 (1.4)	1
Vascular disorders	1 (1.4)	1
Ocular AEs suspected to be drug-related Eye pain Blurred vision	1 (1.4) 1 (1.4) 1 (1.4)	2 1 1
Non-ocular AEs suspected to be drug-related Ischemic cerebral infarction	1 (1.4) 1 (1.4)	1 1
Total SAEs	6 (8.3)	10

^a^Described as number of events. The percentages are calculated with respect to N (number of patients)

*AEs*, adverse events; *n*, number of patients with at least one AE

## Discussion

CNV is the most severe ocular complication of PXE [9]. Untreated cases of CNV secondary to PXE progress to central blindness and thus have a poor prognosis [1, 14]. Findings from the PIXEL study, conducted in a real-world setting, showed that the mean VA was maintained at 1-year with ranibizumab 0.5 mg treatment and thereafter, it remained stable until 4-year follow up. The CNV lesions regressed or stabilized with a limited number of ranibizumab 0.5 mg injections. The proportion of eyes with CNV, CNV leakage and retinal hemorrhage reduced compared with that at the initiation of ranibizumab treatment. The proportion of eyes with CNV was reduced to more than half at 4 years, while those with retinal hemorrhage was reduced to nearly a quarter by 2 years and then remained stable up to 4 years. The proportion of patients with CNV leakage was reduced to less than half at the 4-year follow-up. Ranibizumab 0.5 mg was also well tolerated and showed a favorable safety profile with no reported deaths.

In the PIXEL study, the average age of patients at the appearance of first symptoms was 55.5 years, which is consistent with that in previous literature [23, 24, 29, 31]. The average time to bilateralization of CNV between the two eyes was reported as 2.4 years in the PIXEL study, which was comparable to that reported previously (1.5 years) [9].

Although AS-associated CNV can develop secondary to many systemic disorders, PXE is the most common cause [7]. Since PXE is a rare disorder, much of the experience for the treatment of AS-associated CNV emerges from causes other than PXE. Published literature on anti-VEGF treatment for CNV secondary to AS, mainly case series or studies with either a small sample size or short follow-up, report VA gain or stabilization in 85–93% of the study population [25–28]. Tilleul et al. reported long-term effectiveness of ranibizumab in the largest series (35 eyes of 27 patients) with AS-associated CNV of which 40.7% was due to PXE [29]. Stabilization and improvement of VA was noted in 62.9% of 35 eyes (27 patients) over 4 years. The 2-year results from the same study had reported VA stabilization and gain in 85.7% patients [26]. The VA stabilization and gain was limited to 60% in patients with PXE. Results from the PIXEL study are similar to that from the aforementioned study [26, 29], at the 2-year and 4-year follow-ups (86.3% and 68.4%, respectively), for VA gain and stabilization in PXE patients with AS-associated CNV. Tilleul et al. had also concluded from their 4-year-results that it was easy to achieve but difficult to maintain stable VA over the long term with ranibizumab in AS-associated CNV in eyes with higher initial VA [29]. In the PIXEL study, 50% of the patients had received prior treatment, of which 29.2% had received treatment with other anti-VEGF agents. Thus, one-third of the patients were possibly non-responders to anti-VEGFs which could have hindered VA gain over time.

Anatomically, the presence of CNV and CNV leakage assessed by fluorescein angiography decreased during treatment with ranibizumab from the time of first injection through follow-up after 1, 2, and 4 years. The proportion of eyes with CNV at first injection was 68.4% and dropped by more than half at 1-year follow-up (25.0%) and then remained relatively stable at 2 and 4 years of follow-up (33.3% and 32.1%, respectively). The most common reason for retreatment was systematic treatment as per the ranibizumab dosage recommendation (25.5%) and not CNV progression (9.6%). Thus, it can be assumed that the average number of injections needed for control of CNV would actually be less than what was observed in the study as the proportion of eyes with CNV improved at subsequent follow-up visits, highlighting the decreased rate of CNV progression. In addition, the presence of CNV leakage, one of the markers of disease severity, decreased from 39.8% at the first injection to 8.3% at 1 year, 18.3% at 2 years, and 14.3% at 4 years. However, due to the observational nature of the study design, not all patients underwent a fluorescein angiography during their follow-up. Therefore, one should be cautious when interpreting these data as CNV leakage may have been underestimated. In the future, for long-term serial monitoring of CNV activity in patients with PXE, optical coherence tomography angiography (OCT-A) could be a valuable non-invasive tool. OCT-A also holds enormous potential in early detection of CNV [35]. Earlier experience with anti-VEGF treatment in PXE patients supports initiation of treatment early in the disease pathology as it may potentially arrest disease progression at the outset and optimize treatment outcomes [31]. Thus, OCT-A can help in optimizing treatment outcomes by early detection of CNV and monitoring recurrences as well as existing lesions.

In this study, the mean CRT was observed to decrease from the time of the first ranibizumab injection until 6 months, and thereafter remained stable until the 4-year-follow up. Smaller studies [12, 24, 31] in patients with CNV secondary to PXE have also reported reduction in CRT with anti-VEGF (including ranibizumab) treatment. In addition, Tilleul et al. reported stabilization or decrease in macular thickness in almost 50% of the patients receiving ranibizumab treatment for CNV secondary to AS due to different causes including PXE [29].

Ranibizumab is a humanized monoclonal antibody Fab fragment that is designed specifically for ocular use and is approved for the treatment of CNV secondary to AMD [22]. However, off-label use of bevacizumab to treat CNV is quite common in clinical practice. Bhatnagar et al. reported short-term success in stabilizing or improving VA with bevacizumab in PXE patients with AS-associated CNV, but in 50% of patients there was a need for serial repeated injections (mean number of injections: 1.8) within a short span of 6 months [23]. Finger et al. explored bevacizumab treatment in 16 eyes with CNV secondary to PXE for 28 months and reported improved functional and anatomical outcomes with a mean of 6.5 injections over 28 months [31]. In the PIXEL study, VA stabilization was achieved over 4 years with an average of 4.1 injections, which was much lower compared with the above study. The incidence of ocular/nonocular AEs was low and consistent with that observed in the previously reported clinical studies of ranibizumab in patients with nAMD [19, 20, 36–38]. Only one case of cerebral ischemic infarction was suspected to be related to ranibizumab treatment.

To our knowledge, PIXEL is the first study to report the effectiveness and safety of ranibizumab 0.5 mg treatment in a large series of patients with CNV secondary to PXE for a long follow-up duration of 4 years. Despite the rarity of the condition, the study had a robust sample size of 98 eyes from 72 patients. However, we acknowledge that this study had some limitations: the observational design and the real world settings of the study are limitations as data from all patients were not available at each time point; combining retrospective and prospective data imply some flaws even if the study was designed in a manner to both enlarge the study population and obtain long-term real-world data in a rare disease for which no medical treatment is currently approved and long-term data remains unavailable, and the variations in treatment protocols, ocular examinations in this multicenter study, and patient attrition over the 4-year follow-up period add to the study limitations. Indeed, instruments for diagnosis may have differed across the centers and the number of eyes available for observation was much lower at the 4-year follow-up; therefore, results should be interpreted cautiously (*N* = 98 eyes at first injection, *N* = 79 eyes at 1-year follow-up, *N* = 57 eyes at 2 years of follow-up, and *N* = 28 eyes at 4 years of follow-up). Another important limitation of this study is the absence of assessment of macular atrophy that can develop in PXE independent of CNV, resulting in severe vision loss. Finally, the patients were treated at the investigator’s discretion, thus drawing a conclusion on the dosing regimen of ranibizumab is beyond the scope of the study. Comparison across various studies should be done with caution as the study populations vary with respect to study settings, dosing regimens, previous treatment profiles and underlying comorbidities.

In conclusion, the PIXEL study indicated that treatment with ranibizumab 0.5 mg under real-world setting results in stable VA up to 4 years and regression/stabilization of lesions with a limited number of injections in patients with CNV secondary to PXE. Considering the rarity of PXE and the fact that there is no established treatment for this disorder, treatment with ranibizumab offers a promising therapeutic option for patients with CNV secondary to PXE. Further prospective, longer term studies are needed to evaluate the long-term effectiveness and safety of this treatment.
